# The effect of body mass on performance of the Star Excursion Balance Test (SEBT)

**DOI:** 10.1186/1757-1146-8-S1-A16

**Published:** 2015-04-20

**Authors:** Daniel Waddington, Jade Warren, David Diep

**Affiliations:** 1 Department of Bioscience, University of East London, UK

## Background

Obesity is a global health epidemic and considered a pre-requisite to falls due to the effect of increased mass on balance capability. There is limited research on the effect of increased mass on dynamic balance. The narrow scope of research that does exist assesses balance with sophisticated instruments which lack transferability to the clinical setting. The SEBT is a common clinical tool which has proven to be reliable and sensitive in detecting chronic ankle instability and risk of lower limb injury in athletes. To date, no studies have implemented the SEBT in analysing the effect of obesity or increased mass on balance. Therefore, the aim of this study is to investigate the effects of increased body mass on performance of the SEBT.

## Method

Twenty-eight healthy participants were included in the study: 9 Male, 19 Female; mean (SD): age = 25.5 (5.1) years; height = 1.68m (0.09); mass = 67.8kg (13.6); leg length = 89.0cm (5.6). After four practice trials, participant’s performance was evaluated for three complete trials of the SEBT and a further three with an empathy suit applied. The gender-specific empathy suits were used to increase the participant’s mass, represent the distribution of adipose tissue and the associated limitations experienced in obesity. Reach distances were standardised by the participant’s leg length and the deficit calculated by deducting the Standardised Maximum Reach while wearing the empathy suit from the Standardised Maximum Reach while not wearing the empathy suit. Paired T-Tests were used to analyse the reach deficits.

## Results

All participants demonstrated a reduction in reach distance whilst wearing the empathy suits compared to when the empathy suits were not applied. The reach deficits were significant in seven of the eight reach distances. The anterior reach deficit was found to be the most statistically significant when participants mass was increased (p=0.00002).

## Conclusions

The SEBT has found to be effective in detecting reach deficits related to increased body mass; as applied by gender-specific empathy suits. This indicates its potential use as a clinical tool to quantify dynamic balance ability.

